# The Person-Based Approach to Intervention Development: Application to Digital Health-Related Behavior Change Interventions

**DOI:** 10.2196/jmir.4055

**Published:** 2015-01-30

**Authors:** Lucy Yardley, Leanne Morrison, Katherine Bradbury, Ingrid Muller

**Affiliations:** ^1^Department of PsychologyFaculty of Social and Human SciencesUniversity of SouthamptonSouthamptonUnited Kingdom; ^2^Primary Care and Population SciencesSchool of MedicineUniversity of SouthamptonSouthamptonUnited Kingdom

**Keywords:** person-based approach, Internet, qualitative research, evaluation studies, feasibility studies, health promotion, patient education, professional education, behavior change.

## Abstract

This paper describes an approach that we have evolved for developing successful digital interventions to help people manage their health or illness. We refer to this as the “person-based” approach to highlight the focus on understanding and accommodating the perspectives of the people who will use the intervention. While all intervention designers seek to elicit and incorporate the views of target users in a variety of ways, the person-based approach offers a distinctive and systematic means of addressing the user experience of intended behavior change techniques in particular and can enhance the use of theory-based and evidence-based approaches to intervention development. There are two key elements to the person-based approach. The first is a developmental process involving qualitative research with a wide range of people from the target user populations, carried out at every stage of intervention development, from planning to feasibility testing and implementation. This process goes beyond assessing acceptability, usability, and satisfaction, allowing the intervention designers to build a deep understanding of the psychosocial context of users and their views of the behavioral elements of the intervention. Insights from this process can be used to anticipate and interpret intervention usage and outcomes, and most importantly to modify the intervention to make it more persuasive, feasible, and relevant to users. The second element of the person-based approach is to identify “guiding principles” that can inspire and inform the intervention development by highlighting the distinctive ways that the intervention will address key context-specific behavioral issues. This paper describes how to implement the person-based approach, illustrating the process with examples of the insights gained from our experience of carrying out over a thousand interviews with users, while developing public health and illness management interventions that have proven effective in trials involving tens of thousands of users.

## Introduction

### Overview

This tutorial paper is intended as an introduction to the person-based approach to intervention development. The first section explains how the person-based approach can contribute to effective intervention development and considers how it relates to other approaches. The second section describes how the person-based approach can be implemented throughout intervention development, illustrating its use with examples from our own development of successful interventions. Finally, we present a set of person-based intervention features that are likely to improve acceptability and engagement in most digital interventions.

### Aims and Background of the Person-Based Approach

The fundamental aim of the person-based approach is to ground the development of behavior change interventions in a profound understanding of the perspective and psychosocial context of the people who will use them, gained through iterative in-depth qualitative research. There is widespread consensus in the eHealth research community that eliciting and addressing the needs and perspective of the intended intervention user is a vital part of good intervention development [1-3] to ensure (at a minimum) that interventions are usable and engaging. This is a critical issue for eHealth if it is to fulfil its potential and overcome the problems of low uptake and adherence [4]. It is difficult even for expert intervention developers to fully anticipate the priorities and needs of users [5], and so intervention developers already routinely elicit the views of target users in a variety of ways [6,7], but there is surprisingly little debate and detailed guidance concerning how best to do this [8]. The person-based approach provides a process that enables developers to gain vital insights into how different people experience and implement interventions, and a framework to help developers identify the key characteristics that will make an intervention more meaningful, attractive, and useful to those who engage with it.

The person-based approach was developed by our research team through practical experience of creating and evaluating numerous successful health-related interventions, including public health interventions (eg, to manage weight and stress, promote physical activity and hand hygiene) and illness management interventions (to help users cope with dizziness, back pain, fatigue, respiratory conditions, hypertension, diabetes, cancer, stroke, and many more health problems). Hence, the person-based approach is based on over a thousand in-depth interviews to understand users’ needs and elicit reactions to our interventions [9-16]. Trials of these interventions in tens of thousands of users have demonstrated that what we have learned has proved effective in practice [17-22]. We therefore feel that it is timely to communicate what we have learned to date and explain the methods we have found useful to understand and respond to the perspectives of people who use our interventions. There are many good routes to developing effective interventions and we do not claim that our approach is the only one, but we believe that new intervention developers will find this guide a useful starting point. Given the limited discussion to date of how the user perspective should be incorporated into intervention development, this paper may also stimulate productive debate among more experienced intervention developers regarding the relative advantages and limitations of alternative ways of incorporating the user perspective.

The person-based approach is not intended to replace but to complement and enrich the well-known “theory-based” and “evidence-based” approaches to incorporating behavioral science into intervention development [23,24]. Theory-based approaches have provided valuable frameworks and models for anticipating and describing the likely influences on behavior [25-27], which can then be mapped onto appropriate behavior change techniques [28]. However, a complementary approach is needed to understand the most effective way of *applying* these models and techniques in the specific context of each intervention and the individuals who will use it. The person-based approach yields vital insights into how different people in different situations perceive and execute the behavioral elements of the intervention, why some elements may be particularly necessary or salient to them—or alternatively may be aversive or problematic—and thus how the intervention can be made more attractive, persuasive, and feasible to implement.

### Wider Context of the Person-Based Approach

We refer to this approach as the “person-based” approach to highlight the focus on understanding and accommodating the perspectives of the people who will use the intervention, which we consider essential to maximizing the acceptability and effectiveness of interventions. We use the term “person” rather than “user” or “human” to distinguish our approach from related but somewhat different approaches (see next section for discussion). The generic term “person-centered” was chosen rather than “patient-centered” since the relevant people often include health professionals or healthy members of the community. The term “person-centered” has traditionally been used principally in the context of the person-centered approach to psychological therapy first advocated by Carl Rogers [29]. Although our approach to person-based intervention development was not derived from Rogerian person-centered therapy, it shares the Rogerian emphasis on respect for the autonomy and empathic understanding of the person the intervention is designed for. The term person-centered also has a history in the ethics of respect for personal autonomy in health care [30], which again is consistent with our approach.

The person-based approach to intervention development is not intended to be relevant only to digital interventions, and we have successfully used it to develop offline interventions [31]. However, we consider it particularly relevant to developing digital interventions because people typically use these independently, and so they must be designed with an understanding of how people do this. In addition, the emphasis on autonomy in the person-based approach is consistent with the widely held belief that the Internet can and should be a medium for empowering users [32,33].

The person-based approach has similarities to the use of qualitative methods to elicit user views in “usability testing” [34]. Usability testing is employed in the development of a product (often as a part of user-centered design) to ensure that the product is easy to use and fit for purpose. Usability testing can involve various kinds of qualitative data collection (eg, focus groups, observation, expert panels, interviews, and think aloud studies) at various stages of intervention development (eg, needs and context assessment, building and validating the intervention, real-world use of the intervention) [7]. However, usability testing evolved in the disciplines of human-computer interaction and system design and consequently focuses principally on the technological format of the intervention and the extent that users find it easy and attractive to use and effective for the tasks they need to perform. There are also points of convergence between the person-based approach and the evaluation of dimensions of the user perspective such as acceptability, engagement, trust, and satisfaction [35-37]. But whereas ensuring usability, acceptability, and satisfaction are necessary and important objectives, the goals of the person-based approach are more wide-ranging—to ensure that interventions are also motivating, enjoyable, informative, convincing, and most importantly that they change behavior and/or enhance well-being.

Although the person-based approach is not restricted to the development of digital interventions, it is highly compatible with the more in-depth approaches that have evolved within the disciplines of information systems and human computer interaction, such as human-centered and user-centered design [6,38,39]. These approaches seek to understand the user’s knowledge, skills, behavior, motivations, cultural background, and organizational context, and they involve users iteratively throughout development [3,6,40]. However, the person-based approach is rooted within the discipline of health psychology and is intended for application to developing health-related behavior change interventions that may or may not include a digital product. Consequently, the person-based approach focuses primarily on the behavior change techniques the intervention is intended to deliver, and their implementation by the people using the intervention, including when they are not online. For example, when eliciting user views we specifically direct our participants to give their reactions not to the webpages or screens but to the intervention content, for example, the arguments made and activities suggested and the barriers they have encountered trying to follow the intervention advice (see next section for more discussion of these methods). Because of this difference in focus, the person-based approach could be usefully incorporated within a multidisciplinary holistic human-centered design framework [3], providing a systematic process for contributing an in-depth health psychology perspective that can be used to help implement the participatory and persuasive design aspects of the framework (see Table 1).

**Table 1:** An overview of how the person-based approach can be incorporated at each stage of the development of digital health-related behavior change interventions.

Intervention stage^a^	Target output of the person-based approach	Specific person-based approach processes undertaken	Activities that may be undertaken as part of wider intervention development context
Planning (months 0-6)	Identification of key behavioral issues, needs, and challenges the intervention must address	Synthesize previous qualitative studies of user experiences of similar interventions	Consultation with experts, members of user groups, other stakeholders (eg, purchasers of health care services)
		Carry out primary qualitative research using open-ended questions to elicit user views of the planned behavior changes (including relevant previous experience, barriers and facilitators)	Examination of relevant theory and evidence from previous trials (complex intervention development)
			Observation of real-life context of intended health care product (user-centered design)
Design (months 3-9)	Creation of guiding principles to help developers summarize and easily refer to features of the intervention identified as central to achieving the intervention objectives	Create guiding principles, comprising: key intervention design objectives (addressing key behavioral issues, needs, challenges identified in Step 1), and key (distinctive) features of the intervention needed to achieve objectives (drawing on intervention planning in Step 1)	Theoretical modeling (complex intervention development) eg, creation of logic model describing hypothesized mechanisms of action of intervention, and/or intervention mapping of behavioral determinants and behavior change techniques
			Creation of personas, scenarios, use cases (user-centered design)
Development and evaluation of acceptability and feasibility (months 6-18)	All intervention components evaluated in detail and optimized from user perspective	Elicit, observe and analyze user reactions to every intervention element (eg, using think-aloud techniques), iteratively modifying intervention to optimize from user perspective	Development of detailed procedures for intervention plus information/advice, manuals, scripts, training, etc, for patients and/or health professionals
		Carry out detailed longitudinal mixed methods case studies to evaluate and optimize independent usage of intervention	Mixed methods evaluation of acceptability, feasibility (complex intervention development)
			Creation and usability testing of prototype product (user-centered design)
Implementation and trialing (starting from months 12-18)	Intervention evaluated in real-life context(s), modified to improve implementation in future contexts	Use mixed methods process analyses to identify further modifications to improve acceptability, feasibility, and effectiveness of intervention for future implementation, or for use in different contexts	Effectiveness and cost-effectiveness evaluated using experimental methods (eg, randomized controlled trials), audits, etc
			Mixed methods process analyses of implementation (reach, fidelity, context effects, etc), mediators, and moderators of intervention effectiveness

^a^Timelines given for each stage are indicative only.

### Overview of the Person-Based Approach to Intervention Development

The first section of this paper describes the process of implementing the person-based approach, explaining how in-depth qualitative research can be used to inform initial intervention planning, to elicit views of intervention elements and materials throughout the intervention development, and to understand usage and outcomes during intervention implementation. We illustrate the process and the insights it can generate using brief examples from the wealth of experience we have accumulated during the development, evaluation, and successful implementation of a range of very different interventions. We also describe how to encapsulate the most important insights from the process in “guiding principles”, which provide a means of specifying and communicating the key objectives and distinctive features of interventions. The second part of the paper then outlines and discusses a set of common guiding principles that have emerged as useful across most of our interventions. This paper can provide only an introduction to the approach; our team will be publishing a series of supplementary papers shortly providing further detail and illustration of how it can be implemented.

## The Process of Incorporating a Person-Based Approach Into Intervention Development

### Core Elements of the Person-Based Approach

The person-based approach is incorporated into intervention development first through in-depth qualitative research with users and then through the development of “guiding principles” that state the key intervention design objectives and describe the key features of the intervention required to achieve each objective. These processes are discussed in detail below and examples from our own intervention development are presented to provide illustration. An overview of the person-based approach is shown in Table 1, which illustrates how the person-based approach can be integrated within the wider context of the activities undertaken during intervention development.

Qualitative research is central to the person-based approach at all stages of intervention development and evaluation, including planning and design, early development, acceptability and feasibility testing, and evaluation in clinical trials and real-life settings. In many cases, the research and development will not move through these stages in a linear fashion but will instead go through development-evaluation-development cycles in order to make improvements to the intervention based on data gained during evaluation phases [23]. The following two sections explain in more detail how qualitative research can be used to inform intervention development in each phase.

### Using Qualitative Research to Inform Intervention Planning

At the planning stage, we regard drawing on theory, evidence, and the perspective of the people who will use the intervention as equally important and complementary. Theory and previous quantitative research can provide insight into the intervention components that have potential to be effective or cost effective but often do not offer clear guidance about which are most important or how best to implement them in a particular context. After completing a rigorous process of intervention mapping or causal modeling, using evidence from existing quantitative primary research, systematic reviews, and meta-analyses, intervention designers are often faced by a bewildering range of behavior change techniques that could be used, with no clear evidence on which to base selection of particular combinations. Qualitative research or expert and user consultation is often deployed to attempt to resolve this problem, but this element of theory-based intervention development is often somewhat ad hoc, piecemeal, and not well articulated. The person-based approach provides an explicit and rigorous process for exploring and analyzing the attitudes, understanding, needs, and situation of the people who will be using the intervention in order to select those intervention components that seem the most acceptable, feasible, and salient to them—and crucially to avoid the inclusion of those that are disliked or seen as impractical or intrusive (Textbox 1; [41]). Exploratory qualitative research at this stage can also suggest the need for intervention components or characteristics that have not been considered or previously used in this context (and hence may not yet be evidence-based).

**An illustration of qualitative research identifying potential intervention features which are unacceptable to users.**

At the planning stage of developing a behavior-change app, we carried out focus groups with smartphone users (N=19) to explore the kinds of app features that were viewed as acceptable or unacceptable [41]. One app feature that computer scientists and behavior change experts have been excited about is context sensing, which enables mobile phones to sense where a person is (eg, near a fast food restaurant) and their likely emotional state, in order to send intelligent notifications to support behavior change at key points (such as if a person becomes stressed). However, we found that participants were skeptical about these intelligent notifications, as they believed that the sensing might be inaccurate. They also felt that even when accurate, context sensing might have a negative effect on behavior change, for instance by pointing out that someone was close to a fast food outlet that they had not previously noticed. Based on this lack of trust, we decided not to use context sensing to detect potential behavior change moments, but instead to use it to try to identify times that users would find it convenient and appropriate to receive notifications.

It may be possible to draw on existing qualitative research to understand the perspective of people who will use the intervention; if sufficient relevant research is completed, it may even be possible to carry out a qualitative synthesis of relevant user views. For example, when developing the POWeR weight management intervention, we carried out a synthesis of qualitative research on experiences of weight management interventions [42]. This suggested that providing regular face-to-face support for weight management might be problematic in the longer term, as it might promote dependency on this support to maintain weight loss. We concluded that brief nurse support for our intervention might be preferable to regular nurse support, and in our feasibility study of implementing POWeR [18] in a primary care setting, long-term outcomes did indeed prove better for those with only brief nurse support.

Often there is little or no high-quality qualitative research that is directly relevant to the specific intervention context (eg, a patient with a particular health condition and a particular intervention approach for that health condition). If there is a lack of qualitative research to provide guidance, then it is useful to carry out your own, in order to fully understand users’ perspectives before designing an intervention (see Textbox 2; [43-45]). It is beyond the scope of this paper to give detailed guidance on how to carry out the very wide range of qualitative methods that can be used, but the examples given provide references to practical illustrations.

**Qualitative research conducted to inform intervention planning.**

When planning an online intervention to promote prudent antibiotic prescribing across Europe, it was unclear which intervention strategies might be most acceptable to general practitioners (GPs). No existing research had explored GPs’ attitudes to the variety of different intervention strategies that aimed to promote more prudent prescribing of antibiotics, and it was unknown whether GPs’ attitudes might vary across Europe countries. We conducted 52 interviews with GPs from several European countries, exploring perceptions and experiences of strategies aimed to reduce unnecessary antibiotic prescribing [43]. GPs valued interventions that allowed discussion of prescribing practices between colleagues and the use of diagnostic tests to ensure appropriate prescribing. GPs’ views were highly consistent across countries, suggesting that a single intervention could be suitable across European countries without requiring significant tailoring for each country. An online intervention was therefore created that supported the use of diagnostic tests and an in-house seminar to facilitate discussions of prescribing between colleagues. This intervention proved very acceptable [44] and successful [45] across six European countries.

Target users may vary in their requirements of an intervention or in their beliefs about their health condition. Views may vary depending on a range of characteristics, such as gender, cultural background, health literacy, or previous experiences. It is therefore vital for intervention developers to consider who they need to talk to, so that they can purposively sample [46] a diverse range of users who vary in characteristics that are considered important. This helps ensure that the researcher has insight into all relevant perspectives, enabling the intervention to be tailored to the different types of people who might use it.

When carrying out interviews to explore user views, it is valuable to use very open-ended questions that allow the respondents flexibility to interpret and answer the question in their own way, for example, asking “How do/did you feel about making this behavior change?”. Using very open questions enables the researcher to capture novel responses that might not have been predicted from theory, or by the intervention developer [47]. Focus groups are also well suited to this kind of exploratory qualitative research since group discussion about a topic can lead in unexpected directions and give lay people confidence to express views that contradict the assumptions of the researcher. Fewer open-ended questions (eg, addressing specific dilemmas relating to intervention design, or mapping onto pre-existing theoretical categories) can be used at a later stage of the interview or focus group to check respondent views of aspects of the intervention that the developer is particularly interested in, or that respondents may not have considered spontaneously. However, there is a risk that using questions that ask specifically about particular topics may lead participants to express views of dimensions of the intervention that do not matter greatly to them, making it harder for the intervention developer to distinguish what is really important to prospective users. See Textbox 3 [48-50].

**An illustration of qualitative research to inform intervention planning.**

In the early stages of planning an exercise intervention to reduce the risk of falling in older people, we carried out focus groups and interviews. The interview questions (which were based on the Theory of Planned Behavior) asked specifically about the perceived advantages and disadvantages of carrying out fall prevention exercises and how easy or difficult this would be to do. This study generated an extensive list of potentially relevant barriers and facilitators to uptake and adherence [48]. The focus groups asked more open questions about people’s experiences and views of falls prevention. It was this study that most clearly revealed the crucial barrier—that almost all older people saw falling as meaning that they were becoming frail and dependent, and therefore they completely rejected the idea that they were at risk of falling or had any need of a falls prevention intervention [49]. This insight allowed us to identify the core characteristic of an acceptable intervention as being a positive approach to improving balance in order to maintain fitness and independence, rather than preventing falling [50].

### Creating Guiding Principles to Guide Intervention Design

During the intervention planning phase, it is useful to produce guiding principles that can be consulted throughout the planning and development phases to ensure that a coherent focus underpins the intervention. The guiding principles consist of two elements: (1) intervention design objectives, and (2) key features of the intervention that can achieve these aims. The intervention design objectives articulate the intention to address the key context-specific behavioral needs, issues, or challenges that have been identified during the planning stage. By also then summarizing the key features of the intervention that will achieve these objectives, the guiding principles succinctly capture the characteristics of the intervention that should optimize its acceptability, feasibility, and therefore effectiveness.

The guiding principles are not intended to be exhaustive and do not replace the more extensive documentation of how the elements of the theoretical model underpinning the intervention map onto the behavior change techniques used. Instead, they are intended to complement this detailed planning by helping developers to summarize and easily recall and refer to features of the intervention that intervention planning has identified as central to achieving the intervention objectives. Hence, intervention planning provides a complete and generalizable map of all the generic behavior change elements in an intervention, whereas the guiding principles highlight the distinctive and particular qualities of the intervention—how it seeks to solve the challenges that the intervention addresses in ways that differ from previous interventions.

To provide the context for the guiding principles, it is useful to first clearly state the intervention objectives, in terms of behavior change and outcomes. It is also useful to briefly describe the key characteristics of the target users of the intervention, in terms of their psychosocial characteristics and the behavioral context in which they will be using the intervention (eg, motivation to change their behavior or potential barriers to using certain intervention components). This process can be considered analogous to systems design approaches that create “personas” and “scenarios” [51] but places more emphasis on the psychological aspects of the user and their context. Textbox 4 and Table 2 outline the process of creating guiding principles.

**Illustration of the process of creating guiding principles.**

Context of intervention:

1. State objectives of the intervention, in terms of behavior and outcomes

For example: To support users of exercise referral schemes to increase their physical activity levels (minutes per week) for at least 12 months.

2. Briefly describe relevant aspects of users and their context

For example: People with long-term health conditions referred to an exercise referral scheme, which will provide human support for increasing physical activity. May have limited education and familiarity with computers, low baseline levels of activity, limited confidence, skills, and motivation to undertake activity.

3. Identify key behavioral issues, needs, or challenges the intervention must address.

For example: Currently low long-term adherence to activity in people referred to schemes. Qualitative research links this to barriers to undertaking facility-based education (cost, travel, time, dislike of social environment in facilities).

Create guiding principles (see Table 2):

describe key intervention design objectives

describe key features of the intervention needed to achieve objective

**Table 2:** Creating guiding principles.

Intervention design objectives	Key features
To help people to maintain their activity independently	Digital intervention to build autonomous motivation, self-regulation skills (eg, graded goal setting and self-monitoring) and confidence to become their own physical activity coach.
To help people maintain exercise in the long term	Focus on creating sustainable lifestyle physical activity habits rather than relying on supervised facility-based activities.
To reassure people with long-term health conditions that exercise is safe for them	Encouragement and reassurance for undertaking physical activity with long-term conditions provided in terms of condition-specific advice on consequences of activity, modeling examples of others with similar health conditions, links to local in-person support (including exercise referral scheme).

The first stage of creating the guiding principles themselves is to formulate the intervention design objectives, which serve to focus the developers’ attention on the need to address these issues. For example, qualitative development work for our PRIMIT hand hygiene intervention [13] revealed that most people felt that they already washed their hands often enough; a primary aim of the intervention was therefore to convince people that washing their hands more often was necessary and beneficial [13]. Second, for each intervention design objective we identify some key features of the intervention needed to achieve the aim (Textbox 5; [11]). The intervention planning process may suggest appropriate behavior change techniques or other intervention elements. Note that the focus and content of the principles can vary greatly since the relevance of different aspects of the intervention content and delivery depend on the intervention context and the behavioral issues identified. For example, the key features might include a characteristic relevant to the technology used (such as providing only very brief intervention modules if mobile phones will be used for delivery), or perhaps the implementation setting (eg, embedding the intervention within primary care).

**An illustration of creating guiding principles.**

Our online weight management intervention (POWeR) was created for obese adults, to be implemented in primary care. A key objective of POWeR was for it to produce sustainable weight loss, which could be maintained in the long term.

Qualitative research conducted at the planning stage was extremely useful in helping us to understand our users’ needs [11]. Our inductive interviews (N=25) highlighted that overweight adults:

- had experienced multiple previous failed attempts at dieting
- attributed previous unsuccessful weight loss attempts to feelings of deprivation and regimes that disrupted their lifestyle, were effortful and unsustainable (like calorie counting)

We therefore decided on two main intervention design objectives:

- to persuade users that the POWeR approach to weight management will be effective
- to promote long-term adherence and maintenance of weight loss

The key intervention features chosen to achieve our aims are shown below.

Key features to persuade users that the POWeR approach to weight management will be effective:

- a distinctively different approach containing new, surprising, and interesting content, eg, “POWeR tools” (self-regulation techniques)
- explicitly evidence-based, presenting scientific rationale for recommendations and proof of their effectiveness
- trusted and credible sources; developed by named team of medical and behavior change experts, non-commercial, linked to their own primary care team

Key features to promote long-term adherence and maintenance of weight loss:

- emphasis on building autonomous motivation, non-prescriptive approach (eg, no forbidden foods, choice of eating plans and goals)
- focus on creating lifestyle-compatible long-term habits (less reliance on conscious self-regulation through calorie counting, diary keeping)
- self-efficacy and positive affect promoted by encouraging and rewarding achievable goals, modeling overcoming barriers using engaging stories

### Using Qualitative Research During Intervention Development, Evaluation, and Implementation

Once an intervention has been fully planned and a prototype version created, further qualitative research is essential to gain insight into whether the intervention is acceptable, interesting, persuasive, easy to use, and feasible for people to adhere to. Think-aloud interviews are particularly useful as they ask people to give their immediate reactions to every element of the intervention and allow the researcher to also observe how it is used [52]. As user feedback is gained, changes can be made to the intervention and then further interviews can be conducted to check whether the changes made are suitable. The development phase is therefore best viewed as an iterative cycle moving between user feedback and changes to the intervention.

It is important to note that this process is different from co-design with members of the target population. Sometimes developers seek the opinions of users concerning what elements and characteristics they believe the intervention should include. A potential problem with this approach is that it encourages users to try to anticipate the needs of others, which they are unlikely to do well, rather than simply reporting their own experiences and views, which they do very well. We find that users are naturally expert at telling us what they like or dislike about our intervention, but most users are understandably less able to generate effective behavior change techniques or good design solutions.

It can sometimes be difficult to decide when to implement a change based on user feedback and when not to. If feedback indicates that a feature is potentially off-putting, then it seems prudent to make improvements to the feature. Equally, if feedback reveals participant beliefs that are inconsistent with intervention content, then it makes sense to address these beliefs in the intervention. The guiding principles can be consulted to ensure that any changes implemented are consistent with these principles (Textbox 6; [11]). There are sometimes instances where practical constraints limit the ability for user feedback to be implemented. For instance, in the development phase of a decision-making tool for antibiotic prescribing, a few GPs wanted information specific to their GP practice to be incorporated into the tool, but that level of tailoring for individual practices was not feasible, so this change was not implemented [53].

**An illustration of making intervention modifications based on guiding principles.**

In the development phase of POWeR (our online weight loss intervention), some participants were concerned at the absence of calorie counting in the intervention, as they were used to this approach in previous diets they had tried (and failed to maintain) [11]. However, calorie counting was not consistent with our core objective of promoting long-term maintenance of weight loss by creating healthy habits, rather than using onerous and intrusive self-regulation techniques. Consequently, we chose instead to explain at greater length the rationale for POWeR’s use of alternatives to calorie counting (eg, making simple changes to eating habits and using weekly weighing to monitor the success of these). We also included calorie counting as an aid that could be used briefly to “diagnose” where it might be possible and beneficial to change eating habits.

After a prototype intervention has been refined with feedback from think-aloud interview data, it is useful to ask people to try out using the intervention on their own and then afterwards interview them about their experiences. This provides insight into how people perceive and use an intervention when alone, which might be different from when a researcher is present [54]. This evaluation stage also allows participants to try out behavioral changes and provide feedback about how well the intervention supported the changes they tried. It is helpful for participants to keep a diary of aspects of the intervention that they found helpful or unhelpful, easy or hard to use, and elements they particularly liked or disliked. This can then be drawn on during retrospective interviews to aid participants in discussing aspects that were pertinent to them.

Further intervention modifications may be suggested by the findings of feasibility studies, full scale trials, or when implementing interventions “in the wild”. Including qualitative studies can be extremely valuable in trial and implementation settings, where they are now recommended as part of the wider mixed methods evaluation of implementation [55]. In the implementation context, qualitative research can provide detailed information about how participants experience the intervention and allow exploration of factors or processes that might be involved in adherence to or outcomes from the intervention, enabling researchers to further optimize the intervention (Textbox 7; [56]).

**An illustration of the use of a qualitative study in a feasibility trial.**

We designed an online intervention (SPaCE) to support parents and caregivers of young children with mild to moderate eczema. Qualitative interviews carried out in the context of our feasibility trial of SPaCE revealed that the majority of participants who received health care practitioner support in addition to the website did not find it more helpful than the website alone [56]. As the health care practitioner support was not highly valued and as trial findings showed it did not lead to better outcomes, support was therefore removed from SPaCE for the main trial.

### Practical Considerations

One potential problem that researchers face when carrying out changes to their interventions in the development phase or following evaluation phases is the potential for changes to be costly. This sometimes means that development time can become neglected or squeezed in order to save money. This is particularly the case if computer programming or web design input must be purchased, which is often expensive, meaning that multiple iterations of changes to a digital intervention can become too costly for a researcher’s budget. One solution is to create prototype versions of pages on paper (sometimes referred to by website developers as wireframes), with each page representing a screen to be shown in the intervention (eg, [53]). Some teams develop close collaborations with programmers and flexible in-house software, which makes iterative modifications of interventions less costly and time-consuming [57]. Our solution is to use LifeGuide, an open-source software platform developed by the University of Southampton that allows researchers to change intervention design and content during the development phase [58]. These changes can be made quickly and easily by the behavioral researcher, who does not need previous programming experience. However, we recognize that due to constraints on resources, it is not always feasible to implement the person-based approach fully or at every stage of intervention planning, development, and evaluation.

## Common Person-Based Guiding Principles

In the previous section we outlined the process of using in-depth qualitative research throughout all stages of intervention development and evaluation. We also outlined how qualitative research during the early stages of intervention planning (coupled with insights from existing theory and evidence) can be used to inform guiding principles that identify the key design objectives and key features of the planned intervention. Through our experience of intervention development, we have accumulated a set of person-based intervention features that appear to improve acceptability and engagement in most digital interventions. The following sections describe these objectives and features, which are summarized as common guiding principles in Table 3. These common guiding principles do not provide an exhaustive or prescriptive list of the desirable qualities of an intervention but illustrate common insights that arose from the person-based development process.

Typically, users of digital interventions must feel motivated and confident to use the intervention on their own, and so we have found self-determination theory [59,60] particularly relevant to understanding how users respond to our interventions. The intervention features included within our common guiding principles have therefore been organized under three design objectives relevant to the constructs of self-determination theory. Self-determination theory predicts that intrinsic motivation to engage with health behavior change will be enhanced by supporting users’ need for autonomy (ie, feeling self-directed), increasing users’ sense of competence (control and confidence), and enhancing users’ perceived relatedness or support from the intervention. These three objectives are closely linked and interdependent and are therefore addressed by many of the key features outlined below.

**Table 3:** Guiding principles common to many interventions.

Intervention design objective	Key intervention features
To promote user autonomy	Offering users choice where possible (eg, of goals, tools, timing, method of implementation)
To promote user competence	Providing clear structure and (optional) guidance, examples, stories modeling successfully overcoming barriers, graded goal-setting, minimizing conscious effort and lifestyle disruption where possible
To promote a positive emotional experience and sense of relatedness	Using positive (autonomy-supportive) language throughout, giving rationale for advice, acknowledging and addressing concerns
	Ensuring all communications provide something interesting, enjoyable, relevant, and helpful for the user
	Reciprocating intervention usage by providing immediately rewarding feedback
	Following best practice to maximize accessibility, usability, and trust

### Promoting Autonomy

In all of our interventions, we seek to enhance intrinsic motivation (for both intervention usage and engaging with health behavior change) by support users’ need for autonomy. We consider supporting users’ autonomy to be vital for all our interventions because it can encourage users to internalize the advice and behavioral strategies we provide, enabling them to follow and implement these over the longer term and empowering them to become their own health coach [11,61]. Paradoxically, this could result in lower usage of the intervention itself as users become less reliant on an extrinsic guide for their behavioral choices. Promoting autonomy can be challenging, since the very purpose of an intervention and the behavior change techniques it provides are to offer extrinsic support and guidance. We therefore support autonomy by offering users a choice in how they engage with the intervention and implement the advice provided, including the goals they set, strategies they use, and aspects of the timing, order, and delivery of intervention content [62]. For example, we have found that users are more likely to engage with particular elements of the intervention, such as electronic prompts or notifications, if they have opted to receive them and retain control over when and how frequently they are received [15]. This approach means that although we do use computer-tailoring to ensure that users are not given advice they will definitely see as inappropriate or irrelevant, where possible we allow what has been termed “self-tailoring” [63], that is, user selection of relevant topics, information, or strategies.

There can be a tension between supporting users’ autonomy, while still providing clear guidance on how users can best change their behavior. Qualitative research in the intervention development phase is vital for helping us to establish what users are comfortable doing on their own and when clear directives or examples to follow are needed and appreciated. For example, our qualitative piloting of the POWeR weight management intervention showed us that users struggled to select their own appropriate healthy eating goals when asked to fill in blank response boxes. Users were much more successful at setting appropriate healthy eating goals when they were first invited to select a couple of specific, relevant, and achievable goals from a drop-down list of examples before creating one goal of their own choice [11]. Our experience fits with other studies showing that offering too much choice can be overwhelming [64] and that offering complete navigational control can result in lower intervention usage than “tunneling” core intervention content to ensure that users access essential intervention ingredients in a coherent manner (eg, working through motivational, goal-setting, and

implementation planning elements of the intervention in a logical order) [65].

### Promoting Competence

Competence can be promoted by encouraging users to identify changes to their behavior that minimally disrupt their lifestyle and that can more easily evolve into healthy habits that do not rely on continued effortful self-regulation. We also try to ensure as far as possible that physical access to the intervention content fits with users’ daily routines and, where appropriate, typical usage of digital devices. For example, we now design our interventions so that the content can be delivered in smaller bite-sized chunks at convenient moments in order to fit with increasing use of portable devices (eg, smartphones, tablets) as compared to more traditional computer-based “sessions” that require users to allocate a longer block of time.

We also address competence within our interventions by incorporating well-established theory-based behavior change techniques [66], such as graded goal setting (encouraging people to make small achievable behavior change steps to increase their confidence through experiences of success), social modeling of overcoming obstacles, using stories or testimonials from other users (which also increases relatedness), implementation planning [67], and providing tailored feedback based on self-reported progress towards goals (congratulating success in goal achievement and providing remedial advice if goals have not been achieved).

### Promoting a Positive Experience and Relatedness

The way that behavior change techniques are communicated to people undertaking behavior change interventions is crucial to engagement and adherence, influencing how receptive they are to the advice provided. We understand that the behavior change process can be onerous and challenging and that adherence to interventions is often a low priority for users. We therefore place particular emphasis on attempting to provide users with an enjoyable, positive, and interesting experience of the intervention that can motivate intentions to engage with it [68,69]. Described below are a number of strategies that can be used to foster a positive intervention experience.

First, we always introduce our intervention content and behavior change techniques using a positive autonomy-supportive (ie, non-directive) tone that invites rather than instructs or directs users to try a particular tool or technique. Imperatives and words such as “should”, “must”, or even “we/experts recommend” are avoided wherever possible as they imply extrinsic rather than intrinsic motivation. Instead, we provide an explanation of the scientific rationale or supportive evidence and then invite users to decide for themselves whether following the suggestion will be beneficial, which promotes trust in the advice and is less likely to provoke resistance (Textbox 8; [56]).

**Creating a positive intervention experience by using an autonomy-supportive tone.**

In our SPaCE intervention to help parents manage their children’s eczema [56], behavioral suggestions and advice were introduced using phrases like “some people find it helpful to…” or “you *can* try…” that encouraged users to try out the different suggestions for themselves to see what worked best for them. Parents using SPaCE were also invited to take a 2-week challenge to increase their use of emollient moisturizers; to motivate them to do so we provided anecdotes from other people’s experiences of how they got the most out of using emollients for managing their child’s eczema and how they benefited from the 2-week challenge (eg, “I started using an emollient chart when my daughter got a bit older, and it was great for getting her involved in looking after her skin”).

Relatedly, we ensure that feedback uses non-judgmental language at all times, which can also enhance perceptions of relatedness by showing users that they are respected. For example, knowledge quizzes are a core element of “Healthy Living with Diabetes”, an intervention developed for people with lower levels of health literacy to encourage physical activity in people with diabetes (ISRCTN43587048). Feedback on incorrect answers is phrased as “surprise” rather than “wrong” and also reassures users that giving an incorrect answer is alright and perhaps even expected (eg, “it is not surprising that you think that controlling your sugar levels is the most important thing to do in diabetes—in the past, this is what doctors thought too”). Similarly, users of the POWeR weight management intervention who do not make progress toward their goals are first congratulated for persevering or reassured that slow progress is normal before remedial advice is offered (eg, “don’t worry, many people have weeks like this” or “don’t be too hard on yourself! It is important to give yourself credit after you have had a slip, rather than telling yourself off, especially if you have managed to stop at the slip”).

Acknowledging and addressing users’ concerns about using an intervention or implementing its advice can also help to earn users’ trust by showing them that they are understood and listened to. The inductive qualitative research we conduct during the intervention planning and development phases can help to identify the specific concerns, barriers, and misconceptions that users may have about the intervention itself or the behavior change process. The intervention can then be modified so that these concerns are acknowledged and addressed before seeking to change users’ knowledge, attitudes, or behaviors (Textbox 9; [13]).

**Enhancing relatedness by acknowledging and addressing beliefs and concerns.**

Qualitative piloting of the PRIMIT intervention to promote hand hygiene [13] revealed that users were concerned that frequent hand-washing might be obsessive and could lead to dry skin. Many people also believed that respiratory viruses were transmitted entirely by air and not by hand-to-mouth contact. To address concerns about hand-washing, we provided advice on how to use moisturizers to prevent dry skin and modeled frequent hand-washing as considerate and prudent rather than obsessive behavior. We also included at an early stage of the intervention compelling evidence that hand-washing could reduce flu transmission. An FAQ section was added collating answers to other common problems and concerns revealed by our qualitative piloting.

A second key feature of our interventions is to ensure that all communications provide something useful and relevant for the user. In focus groups we ran with young adults to explore their experiences and views of health apps, we discovered that users can be extremely annoyed by email prompts or push notifications and may actually feel harassed by them [15]. We therefore try to ensure that prompts and notifications create a positive experience by incorporating new, additional intervention content that offers something useful or interesting to the individual users at the time it is received [70]. For example, notifications triggered by a smartphone-based intervention for stress management (“Healthy Mind”) (ISRCTN67177737) give users new, interesting facts about a tool in the app before inviting them to actually use it (eg, “did you know that thinking positively can protect your body against stress? That’s because positive emotions lower your heart rate and blood pressure after something stressful happens”).

Similarly, we try to provide our users with some immediately useful and rewarding communication (eg, tailored feedback) in return for any time spent completing intervention activities (eg, self-reflection, self-monitoring, answering knowledge quizzes). Think-aloud studies conducted alongside the development of an intervention to support the self-management of minor stomach and bowel complaints (“Gut Instincts”) showed us that users preferred intervention usage to be a reciprocal interaction [71]. Users’ interest in reflecting on or answering questions about their symptoms, thoughts, or behaviors was maintained only if this was immediately reciprocated by appropriate personalized feedback. Our experiences are consistent with findings from reviews showing that self-monitoring tools are most effective when they also provide feedback on users’ performance or progress [72,73].

Finally, we follow existing best practice for maximizing the accessibility, usability, and credibility of the intervention for a wide range of people, including those with lower levels of literacy [74] or cognitive impairments [75] (eg, using short sentences, list and audio-visual formats, tailoring where appropriate). We follow existing guidance on usability and interface design to ensure that users can navigate and process our interventions as quickly and painlessly as possible (eg, large font size/buttons, minimal scrolling/clicking, consistent page layouts, clear signposts, etc [76,77]). We recognize that users can become overwhelmed or bored if presented with too much information to read, process, and implement in one go. To minimize this, we present only the information that is essential for the user to read at a specific point in time with any additional non-essential information provided as an optional click-through [9].

There is already a wealth of literature and published guidelines that suggest further strategies for enhancing trust in and credibility of interventions [78-82]. These include providing options to receive personally relevant or tailored information; a professional and consistent visual appearance; error free and up-to-date content; usable interface; provision of supporting evidence for the information provided; details and credentials of the team responsible for developing the intervention; providing reassurances about what data are collected from users, how that data will be used in the delivery of the intervention, and how that data will be securely stored; and opportunities to contact and provide feedback to the intervention team (either via the intervention or in a follow-up interview).

## Conclusions

The purpose of the person-based approach is to ground intervention design in a rigorous, in-depth understanding of the psychosocial context of the people who will use the intervention, derived from iterative in-depth qualitative research. This approach can be used in the development of any intervention involving self-management but has particular relevance to optimizing autonomous engagement with digital interventions. In this context, the person-based approach could readily be integrated with and contribute to user-centered design by highlighting the psychosocial issues relevant to the behavioral change process, in addition to the (equally important) issues relating to usability and engagement with technology that are traditionally the principal focus of user-centered design.

The person-based approach can also usefully complement theory-based and evidence-based intervention development; indeed, it seems self-evident that intervention design can benefit greatly from being not only theory-based and evidence-based but also person-based. Theory-based and evidence-based approaches to intervention development provide a comprehensive and generalizable analysis of all the potentially relevant theoretical constructs and behavior change techniques, and evidence for which have proven effective in other contexts. However, the person-based approach is crucial for identifying which intervention design features are likely to be most important in the context of a particular population and intervention and provides sensitive guidance on how to implement them in a way that will be acceptable and persuasive to users.
